# Radiation induced cataracts in interventionalists occupationally exposed to ionising radiation

**DOI:** 10.4102/sajr.v26i1.2495

**Published:** 2022-09-30

**Authors:** André Rose, William I.D. Rae, Margaret A. Sweetlove, Lumko Ngetu, Mohamed A. Benadjaoud, Wayne Marais

**Affiliations:** 1 Center for Health Systems Research and Development, Faculty of Humanities, University of the Free State, Bloemfontein, South Africa; 2 Prince of Wales Hospital, Faculty of Medical Imaging, University of Sydney, Sydney, Australia; 3 Department of Medical Physics, Faculty of Health Sciences, University of the Free State, Bloemfontein, South Africa; 4 Department of Ophthalmology, Faculty of Health, University of the Free State, Bloemfontein, South Africa; 5 Institute for Radiological Protection and Nuclear Safety (IRSN), Fontenay-aux-Roses, France; 6 Department of Radiobiology and Regenerative Medicine (SERAMED), Fontenay-aux-Roses, France

**Keywords:** interventionalists, interventional radiology, ionising radiation, radiation cataracts, radiation protection

## Abstract

**Background:**

Occupational exposure to ionising radiation may have detrimental health effects. Longer and more complex fluoroscopic procedures have placed interventionalists at increased occupational health risks especially for developing cataracts in the radiosensitive lenses of the eyes.

**Objectives:**

This study aimed to determine the prevalence of occupational related cataracts and describe the risk factors for cataracts in occupationally exposed interventionalists compared with unexposed doctors.

**Method:**

A cross-sectional study using multiple methods. A survey was conducted. The radiation workload was determined based on a self-administered questionnaire and dose area product values determined in previous studies. Both groups had slit lamp examinations. The data were analysed analytically using R software version 9.3.

**Results:**

The study included 98 interventionalists. The combined prevalence of posterior sub-capsular (PSC) and cortical cataracts was 18.8% in the exposed and 13.9% in the unexposed group. The prevalence of PSC cataracts in the exposed group was 5.9% and 2.8% in the unexposed group, with an odds ratio (OR) of 2.2 (95% confidence interval [CI]: 0.58; 8.61). Posterior sub-capsular cataracts were more common in the left eye. The increase in cataracts was not statistically significant in the exposed group but is of clinical significance.

**Conclusion:**

The findings are important as they highlight the need for greater vigilance for protecting the radiation healthcare workforce in a developing country setting.

**Contribution:**

The research is the first of its kind in South Africa and Africa and contributes to determining the prevalence in this highly skilled and occupationally vulnerable group.

## Introduction

Ionising radiation (IR) is integral and essential in modern medical diagnostic, prognostic and interventional procedures.^1^ The number of procedures has dramatically increased globally over the past few decades.^1^ The technology has improved and lower radiation doses are delivered to patients. Interventionalists are, however, performing more complex procedures, which are lengthier and they are thus occupationally exposed to IR for a longer duration and their eyes are at particular risk of developing cataracts in the long term.^2^ Interventional clinicians such as interventional radiologists, interventional cardiologists and radiation healthcare workers (HCWs) are at high risk of radiation exposure in the catheterisation laboratory.^3^ When compared with other interventional procedures, cardiac catheterisation procedures expose operators to radiation doses 2–3 orders of magnitude greater.^4^ Interventional radiologists and interventional cardiologists receive similar radiation doses in the catheterisation laboratory (even though the procedures are in some ways quite different) and therefore should be similarly trained and protected to mitigate the risk.^2^

The effects of IR on interventionalists include stochastic effects such as cancer and chromosomal aberrations.^5^ It was previously thought that the relationship between IR exposure and cataractogenesis was deterministic, but increasingly there is uncertainty about a threshold level and evidence is mounting that the effects may be evident even at low doses.^6,7,8^ The additive effect of low dose radiation on other cataract risk factors also remains to be answered.^7^ Low dose exposure to IR places radiation HCWs at increased risk of developing cataracts if they are not adequately protected.^9^ The lenses of the eyes are highly radiosensitive and there is a strong correlation between occupational radiation exposure and cataracts.^8^

Cataracts related to occupational radiation exposure are frequently reported to occur in the posterior sub-capsular (PSC) region of the lens of the eye but recent data suggest that it may also occur in the cortical region.^10^ Radiation-induced cataracts also occur more commonly in the left eye compared with the right eye, and this is related to the position in which the interventional cardiologist is working with respect to the X-ray beam.^10^ In a French study conducted between 2009 and 2011, it was shown that cardiologists with a mean age of 51 ± 7.3 had a prevalence of PSC cataracts of 17% (*N* = 109; confidence interval [CI]: 10% – 24%; odds ratio [OR]: 3.8 [1.3–11.4]).^11^ In another study conducted in Malaysia in 2009 the prevalence of PSC cataracts was reported as 54% (*N* = 56; CI: 35–73; relative risk of 5.7 [CI: 1.5–22]). In this cohort there were 56 interventional cardiologists with a mean age of 43 ± 7 years (31–64).^12^

In contrast, separate Greek and Finnish studies showed that there was no statistically significant difference between cataract findings in interventionalists occupationally exposed to IR and a group of doctors not occupationally exposed to IR.^13,14^ In the Greek study, Thrapsanioti, et al. (2017), included 44 interventional cardiologists.^13^ In the Finnish study by Auvinen, et al. (2015) PSC cataracts were detected in 3/21 exposed participants compared with 1/15 unexposed participants (the prevalence ratio was 2.29 [CI: 0.29–19.97] for the exposed group) and the mean age was 54.^14^ It is, however, difficult to compare studies on the prevalence of occupational radiation-induced cataracts as these studies used different grading systems, different assessments of risk factors and there are concerns about dosimetry because of dose uncertainties.^6^ However, these discrepancies do not negate the clinical significance of these studies and the importance of protecting the eyes of doctors (and other radiation HCWs) in this occupational setting.

This is particularly important given the mounting evidence of the detrimental biological effects of low dose radiation to the eyes, which has resulted in the International Commission on Radiological Protection (ICRP) revising its exposure limit recommendations from 150 mSv per year to 20 mSv per year, averaged over five years, with no one year exceeding 50 mSv.^15^ A survey done in the United Kingdom by Public Health England in 2012/2013 found that compliance with these recommendations would be possible in the United Kingdom.^16^ These recommendations, however, have potentially major implications for resource constrained environments such as South Africa (and Africa). In such settings the implementation, control and monitoring of regulatory structures would be a challenge, making it difficult to comply with ICRP recommendations to reduce the dose as mentioned here.^17^

South Africa (and other low- to middle-income countries) has a paucity of highly trained doctors such as interventionalists, which is compounded by an escalating burden of non-communicable diseases that requires these skills for its management.^18^ It is thus important that this human resource is protected and that safety in the workplace is optimised. This can be achieved through several initiatives such as measuring and monitoring IR exposure in the workplace,^19^ enforcing personal dosimetry utilisation and feedback, promoting informed decision making when using imaging in clinical practice,^20^ appropriate use of imaging equipment,^20^ encouraging consistent and appropriate use of personal protective equipment (PPE),^21^ formalised training and continued medical education on radiation safety,^22^ and engaging hospital management structures to support all aspects of promoting radiation safety in the workplace.^23^ Underpinning these initiatives is the creation of a culture of radiation protection (CRP).^23^ This CRP is the cornerstone of the norms, values and standards within an organisation.^24^ The aim of this study was to determine the prevalence of occupational related cataracts and describe the risk factors for cataracts in this study population of occupationally exposed interventionalists compared with an occupationally unexposed group of doctors in South Africa.

## Methods

### Study design

This was a prospective cross-sectional study that formed part of a larger multiple methods study.^25^

### Study population

**FIGURE 1:**
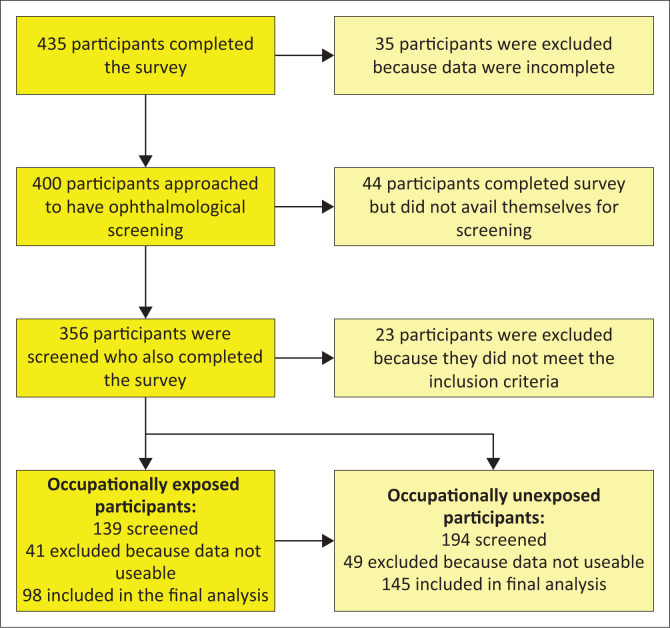
Illustration of the study population that were screened and completed the survey.

Figure 1 illustrates the participants who were recruited for the study. The inclusion criteria for the occupationally exposed participants were interventional radiologists and interventional cardiologists. The occupationally unexposed participants had to be a doctor who was not routinely occupationally exposed to IR. All participants had to have completed the survey and had a slit lamp examination. Participants were excluded because they did not meet the inclusion criteria, because the survey was not fully completed or the data provided were not useable or they did not have a bio-microscopy slit lamp examination. The participants not occupationally exposed to IR included family physicians, specialists physicians, psychiatrists and pathologists. The occupationally exposed participants included 25 interventional radiologists, 42 adult cardiologists and 31 paediatric cardiologists. The two groups were comparable to each other in terms of socio-demographics and levels of education.

### Data collection

Data were collected at conferences and workshops across South Africa between May 2015 and March 2017. The survey was conducted using a paper-based system and an electronic format. The questionnaire collected demographic data, medical risk factors, non-occupational exposure, occupational workload, personal protective equipment utilisation, dosimetry practice and radiation safety training.^25^

### Ophthalmological examination

All participants had a bio-microscopy slit lamp examination by the same trained ophthalmologist using the same slit lamp. The clinician was not blinded to the participants because screening was conducted at radiology or cardiology conferences. The participants’ eyes were dilated and a bio-microscopy slit lamp examination was conducted.^25^ Cataracts were classified according to the World Health Organization Simplified Cataracts Grading Score (WHOSCGS).^26^ The cataracts were graded as cortical, nuclear or PSC.^26^ Visual acuity was measured using a modified Snellen Chart.

### Workload estimation

Workload was calculated from self-administered questionnaires completed by interventionalists who indicated the type of procedure, the number of procedures per week and the number of years worked with fluoroscopy guided interventional procedures. Average dose area product (DAP) values per procedure were obtained from previous work performed both in the same institution as this study and more widely in South Africa.^27,28^ As DAP reflects not only the dose within the radiation field but also the area of tissue irradiated, it is a better indication of scattered radiation, which is the source of radiation to the eye. The DAP was an average for a specific procedure. This average would have changed with time for the specific procedure and it is a limitation that we did not consider in the calculations. Three categories of modifiers were considered: (1) an attenuation modifier accounting for attenuation afforded by the use of ceiling suspended screens and the frequency of use of these screens; (2) a similar modifier for the use of lead glasses and the frequency of use of these glasses; and (3) an escalating modifier for radial (as opposed to femoral) approach and its frequency of use. The maximum modifying factors were taken from published data.^29^ The modification factor for the ceiling suspended screen and the lead glasses was 0.1 and 2.0 for radial access, which was applied to the calculated lifetime DAP of the participants.^30^

### Statistical analysis

Statistical analyses were performed using R software version 9.3 (R: A language and environment for statistical computing. R Foundation for Statistical Computing, Vienna, Austria, URL https://www.r-project.org).

The comparison of demographics between the participants occupationally exposed to IR and those not occupationally exposed was conducted using the Mann–Whitney U-test and chi-squared test according to the nature of the covariates (continuous and categorical, respectively).

Ordinary logistic regression, adjusted for age, was conducted to analyse and compare the cataracts in the left and right eyes in the two population groups. In order to identify the risk factors associated with cortical and PSC cataracts using the left and right eye scoring within each participant, a mixed effect logistic regression was performed generating ORs and 95% CIs (R-package lme4).

### Ethical considerations

The study was approved by the Health Sciences Ethics Committee of the University of the Free State (ECUFS44/2015). All participants consented to completing the survey and to having a bio-microscopy slit lamp examination.

## Results

Table 1 illustrates the basic demographic data and the risk factors for cataracts in the participants. There were 243 participants in total and 98 were routinely occupationally exposed to IR. We included only participants who both completed the survey and had the bio-microscopy slit lamp examination.

**TABLE 1:** Demographics and risk factors with percentages for categorical variables and mean ± standard error for continuous variables. The *p*-values are for comparison of exposed and unexposed groups.

Covariate	All participants (*N* = 243)		Exposed (*n* = 98)		Unexposed (*n* = 145)		*p*
	Mean	Standard error	Mean	Standard error	Mean	Standard error	
**Age**	46.4	±11.7	45.7	±10.0	46.8	±12.8	0.769
**Gender**							
Male	160	65.8	68	69.4	92	63.4	0.338
Female	83	34.2	30	30.6	53	36.6	
**Years worked**	15.9	±11.8	12.9	±9.6	17.9	±12.7	**0.004**
**Risk factors**							
Smokers	16	6.6	7	7.1	9	6.2	0.773
Years smoking	1.21	±5.3	1.23	±4.8	1.2	±5.6	0.780
Uses alcohol	132	54.3	60	61.2	72	49.7	0.076
Years using alcohol	12.2	±14.6	14.5	±15.3	10.6	±13.8	0.036
Myopia	39	16.0	17	17.3	22	15.2	0.651
Hypertension	29	11.9	3	3.1	26	17.9	**0.000**
Diabetes	11	4.5	2	2.0	9	6.2	0.125
Obesity	14	5.8	3	3.1	11	7.6	0.138
Steroid use	1	0.4	0	-	1	0.7	0.410

Note: Significant *p*-values are set in bold.

There was no statistical difference in demographics and risk factors between the exposed and unexposed groups (except for hypertension), which meant that the two groups were comparable in all respects including age, gender and risk factors. In the analysis, years worked for the exposed group refers to how many years they worked performing fluoroscopy procedures and thus is a measure of their duration of occupational exposure to IR. Years worked in the unexposed group refer to how long they have worked as doctors. This may explain the *p*-value (0.004) in the analysis.

There were 11 (10.2%) participants who reported using lead glasses consistently, 66 (61.1%) reported never using lead glasses and 21 (19.4%) never used ceiling suspended screens. This is consistent with low use of protective lead eyewear in other settings.^31^

Table 2 illustrates that there were no risk factors that were statistically significantly associated with any risk factor for cortical or PSC cataracts for all the participants.

**TABLE 2:** The univariate analysis for cortical and posterior sub-capsular cataracts risk factors in all participants.

Covariate	Cortical			PSC		
	OR	CI	*p*	OR	CI	*p*
Gender	1.4	0.87; 2.33	0.151	0.3	0.04; 2.15	0.224
Year worked	1.0	0.98; 1.02	0.964	1.0	0.96; 1.04	0.970
Smoking	0.7	0.35; 1.50	0.378	0.4	0.14; 1.07	0.061
Alcohol	1.0	0.62; 1.55	0.931	1.3	0.49; 3.28	0.615
Myopia	1.3	0.77; 2.20	0.308	0.6	0.119; 2.765	0.479
Hypertension†	1.1	0.61; 1.85	0.820	-	-	-
Diabetes	2.1	1.00; 4.21	0.044	1.3	0.249; 6.308	0.783
Obesity‡	1.6	0.70; 3.49	0.270	-	-	-

PSC, posterior sub-capsular; OR, odds ratio; CI, confidence interval.

† , There were no participants who had PSC and had hypertension and therefore a univariate analysis could not be run.

‡ , There were insufficient observations to run the model and the analysis predicted perfect failure for PSC.

In Table 3, the combined prevalence of PSC and cortical cataracts was 18.8% in the exposed and 13.9% in the unexposed group. The prevalence of PSC cataracts in the exposed group was 5.9% and 2.8% in the unexposed group, giving an OR of 2.2 (95% CI: 0.58; 8.61). Although the difference between the exposed and unexposed groups for PSC was not statistically significant, it was based on very small numbers of cases and the increase was restricted to the left (and most exposed) eye. The 2.2-fold increase in the exposed group may therefore be of clinical significance.

**TABLE 3:** Description of cataracts after exclusion of participants less than 35 years and less than 5 years’ experience.

Posterior sub capsular	All participants (*N* = 229)		Exposed group (*n* = 85)		Unexposed group (*n* = 144)		OR†	CI	*p*
	*n*	%	*n*	%	*n*	%			
PSC uni- or bi-lateral	9	3.9	5	5.9	4	2.8	2.2	0.58; 8.61	0.244
PSC left eye	9	3.9	5	5.9	4	2.8	2.2	0.58; 8.61	0.244
PSC right eye	3	1.3	1	1.1	2	1.4	1.3	0.10; 16.99	0.836
PSC bilateral	3	1.3	1	1.1	2	1.4	1.3	0.10; 16.99	0.836
**Cortical**									
Cortical uni- or bi-lateral	27	11.8	11	12.9	16	11.1	1.4	0.59; 3.41	0.435
Cortical left eye	26	11.4	11	12.9	15	10.4	1.6	0.65; 3.98	0.300
Cortical right eye	21	9.2	7	8.2	14	9.7	1.1	0.38; 2.96	0.911
Cortical bilateral	20	8.7	7	8.2	13	9.0	1.3	0.43; 3.65	0.676
**Nuclear**									
Nuclear uni- or bi-lateral	69	30.1	21	24.7	48	33.3	0.6	0.33; 1.26	0.200
Nuclear left eye	63	27.5	17	20.0	46	31.9	0.5	0.25; 1.04	0.062
Nuclear right eye	65	28.4	20	23.5	45	31.3	0.7	0.35; 1.33	0.263
Nuclear bilateral	59	25.8	16	18.8	43	29.9	0.5	0.26; 1.09	0.087

PSC, posterior sub-capsular; OR, odds ratio; CI, confidence interval.

† , odds ratio adjusted on age.

In Table 4, we would have expected a pattern showing an increase in risk with age and occupational exposure to IR. Even if significant risk of PSC and cortical cataracts was found amongst the exposed practitioners with career duration less than five years and between 11 and 20 years, respectively, a global risk trend was not demonstrated. The correlation between years exposed to IR and cataract was not demonstrated. On the other hand, the age was confirmed as a major risk factor in both types of cataracts increasing the odds by 6% – 7% for each additional age year.

**TABLE 4:** Posterior sub-capsular and cortical cataracts according to career after excluding participants < 35 years of age and years of occupational exposure to ionising radiation.

Covariate	Estimated parameter	s.e.	OR	CI	*p*
**PSC**					
Unexposed (*n* = 144)	1.00	-	1.00	1.00; 1.00	Ref
0–5 years (*n* = 27)	1.56	0.74	4.77	1.12; 20.42	**0**.**04**
6–10 years (*n* = 25)	0.55	0.99	1.73	0.25; 12.10	0.58
11–20 years (*n* = 28)	1.08	0.78	2.95	0.64; 13.66	0.17
> 20 years (*n* = 17)	−3.8	0.92	0.69	0.11; 4.17	0.68
Age	0.06	0.03	1.06	1.00; 1.12	0.04
**Cortical cataracts**					
Unexposed (*n* = 144)	1.00	-	1.00	1.00; 1.00	Ref
0–5 years (*n* = 27)	0.90	0.54	2.46	0.85; 7.16	0.10
6–10 years (*n* = 25)	−0.33	0.78	0.72	0.16; 3.36	0.68
11–20 years (*n* = 28)	0.92	0.39	2.52	1.18; 5.36	**0.02**
> 20 years (*n* = 17)	−0.18	0.36	0.83	0.41; 1.67	0.61
Age	0.07	0.02	1.07	1.04; 1.10	0.00

Note: Significant *p*-values are set in bold.

PSC, posterior sub-capsular; OR, odds ratio; CI, confidence interval; s.e., standard error.

Table 5 demonstrates the years worked with fluoroscopy and the lifetime workload exposure when lead suspended ceiling screens, lead glasses and radial access are considered. The lifetime workload exposure is the cumulative ionising dose that interventionalists were exposed to during their career.

**TABLE 5:** Estimated radiation workload exposure in Gy.cm^2^ for each category of worker without PPE protection and with the different PPE used.

Covariate	Radiologists	Cardiologists	Paediatric cardiologists
**Without PPE**			
Average	289 384	929 813	50 027
Min	3910	20 654	3362
Max	1 274 062	3 937 756	134 895
Median	128 579	706 560	43 244
IQR	64 400–244 743	280 968–1 433 320	13 450–77 418
**Ceiling shield**			
Average	151 374	663 126	45 498
Min	3910	2065	336
Max	1 091 194	3 600 420	129 030
Median	117 208	280 968	37 324
IQR	50 830–128 800	81 843–971 520	7820–73 742
**Ceiling shield with glasses**			
Average	133 098	624 601	41 055
Min	408	2065	336
Max	1 091 194	3 600 420	103 349
Median	90 160	234 894	35 972
IQR	47 140–121 440	61 843–870 780	5943–70 771
**Ceiling shield with glasses and radial access**			
Average	137 773	717 223	41 055
Min	408	2065	336
Max	1 091 194	4 320 504	103 349
Median	105 680	291 345	35 972
IQR	54 096–128 579	78 866–1 068 692	5943–70 771

PPE, personal protective equipment; IQR, interquartile range; Min, minimum; Max, maximum.

## Discussion

The exposed and unexposed groups were both doctors and thus comparable to each other occupationally and socio-economically. In previous studies the control groups were often support staff such as nurses. The comparability of the two groups was further reaffirmed when adjusting for confounders which did not change the results.

The bio-microscopy slit lamp examination was carried out by the same ophthalmologist (L.N.). The advantage of using a single ophthalmologist is that it does not introduce inter-observer bias. The grading was carried out according to the WHOSCGS grading system.^26^ This is a standardised system cataract grading system, which is freely available. This, however, does make it difficult to compare the findings to studies that used a different scoring system.

There was no statistically significant difference between the prevalence of cortical and PSC cataracts in the interventionalists occupationally exposed to IR compared with the occupationally unexposed group although 2.2- and 1.4-fold increases were observed, based on the small numbers of cases. This is in contrast to previous studies that mostly demonstrated an increase of 3–5-fold compared with an unexposed group.^11,12,32^ Our findings, however, corroborate those of two other studies that showed a lower prevalence of radiation associated cataracts compared with the preceding studies cited.^13,14^ The combined prevalence of cataracts for both PSC and cortical was 18.8%. There is evidence to suggest that cortical cataracts may also be associated with radiation.^10^ Although there was no statistical difference in the prevalence of PSC cataracts between the occupationally exposed group compared with the unexposed group, PSC cataracts were 2.2 times more likely than in the unexposed group (OR: 2.2; CI: 0.578; 8.611; *p* = 0.244). This is clinically significant and therefore occupationally significant.

This study findings further showed an increase in cataracts in the left eye compared with the right eye. This finding is congruent with current literature which reports that radiation-induced cataracts are more common in the sub-capsular region in the left eye of interventionalists occupationally exposed to IR.^11^

South African interventionalists spend 2–3 days per week in the catheterisation laboratory and thus may have less accumulated occupational exposure to IR than in countries where interventionalists may spend more time in the catheterisation laboratory. We postulate that this may be a reason that the prevalence of PSC cataracts is not as high as reported in previous studies. Another possible reason could have been that the interventionalists were consistently using lead glasses. However, our study showed that only 10.2% of participants consistently used lead glasses and therefore, there must be other factors that could explain the difference between our findings and studies which showed a higher prevalence.

We did not directly measure the radiation dose to the eye and this is a limitation of this study. Future studies should measure the radiation dose to the eye in the South African context. The workload estimates calculated are limited by the many confounders that could affect the radiation workload estimate. The calculations, however, consider those main factors that could have influenced the workload dose estimates. The workload exposures were a lifetime dose exposure estimate, which were extrapolated from a self-completed questionnaire and may have been affected by recall error. The recall error may have affected the reliability of the findings.

The strength of this study is that it is the first to determine the prevalence of cataracts in interventionalists occupationally exposed to IR in a resource constrained African setting. Africa is rapidly acquiring advanced radiological technologies and it is crucial to protect the health workforce that will be operating these machines.

The results do not negate previous findings of a higher prevalence of radiation-induced cataracts. It, however, does support the need for greater vigilance in radiation protection measures for the eye and the need to develop a CRP in the catheterisation laboratory in order to prevent radiation damage to the eyes. A South African study showed an underdeveloped CRP within the South African context especially amongst South African cardiologists.^23^ The use of personal protective eyewear is imperative for protecting the eyes of interventionalists and should be part of a radiation safety culture.^33^ Education and training is key to developing a CRP.^34^ The training programme for interventionalists and especially cardiologists in South Africa requires urgent and decisive intervention to aid developing an entrenched CRP.^22,35^

## Conclusion

Although there was no statistical difference between exposed and unexposed groups, possibly because of the relatively small numbers of subjects included in the study, PSC cataracts were more likely to occur in interventionalists occupationally exposed to IR. Radiation safety measures should be implemented, encouraged and enforced in interventionalists occupationally exposed to IR to mitigate for IR damage to the eyes. Although this study was conducted in South Africa, the recommendations may be transferable to other resource constrained settings in Africa and other low- and middle-income countries.
